# miR-4634 augments the anti-tumor effects of RAD001 and associates well with clinical prognosis of non-small cell lung cancer

**DOI:** 10.1038/s41598-020-70157-0

**Published:** 2020-08-04

**Authors:** Sile Liu, Hongjing Zang, Hongmei Zheng, Weiyuan Wang, Qiuyuan Wen, Yuting Zhan, Yang Yang, Yue Ning, Haihua Wang, Songqing Fan

**Affiliations:** 1 Department of Pathology, The Second Xiangya Hospital, Central South University, Changsha, 410011 Hunan China; 2 Department of Pathology, Xiangya Hospital of Central South University, Changsha, 410011 Hunan China

**Keywords:** Cancer, Drug discovery

## Abstract

MicroRNA (miRNA) is involved in the physiological and pathological processes of various malignancies. In this study, miRNA microarray analysis showed that miR-4634 levels in A549 cells increased significantly after everolimus (RAD001) treatment. Decreased expression of miR-4634 was also found in non-small-cell lung carcinoma (NSCLC) cell lines and patients’ tumors by qPCR. Additionally, a combination of miR-4634 and RAD001 exerted synergistic antitumor efficacy by inhibiting cell proliferation, migration, and colony formation. High expression of miR-4634 was significantly more common in non-cancerous lung tissue than adenocarcinoma or squamous cell carcinoma tissue (72.8%, 45.7%, and 50.9%, respectively; *P* < 0.001). Furthermore, high expression of miR-4634 was found to be more frequent in patients without lymph node metastasis (*P* = 0.037) by in-situ hybridization. Importantly, through univariate and multivariate analysis, high miR-4634 expression was associated with better prognosis of NSCLC patients. In conclusion, miR-4634 may act as a tumor suppressor in NSCLC, and to augment the efficacy of RAD001, co-treatment of miR-4634 and RAD001 might be a potential mTOR-targeted cancer therapy strategy for NSCLC patients. High expression of miR-4634 could be an independent good prognostic biomarker for NSCLC.

## Introduction

Lung cancer is the most diagnosed cancer and the leading cause of cancer-related deaths, both in China and the United States^1,2^. It was reported that 472,142 men and 218,425 women died of lung cancer in China, and there were 774.323 newly diagnosed lung cancer cases in 2018^3^. Non-small-cell lung cancer (NSCLC) accounts for the vast majority of lung cancers, with squamous cell carcinoma (SCC) and adenocarcinoma (ADC) representing the two major histological subtypes of NSCLC^4^. Despite advances for NSCLC therapy in recent years, most patients are diagnosed with NSCLC at advanced stages, when the disease is inoperable with poor prognosis^5,6^. Therefore, there is an urgent need to find an effective indicator to identify NSCLC during the early stages of disease.

MicroRNAs (miRNAs) are a species of small endogenous non-coding RNA of 19–25 nucleotides in length, which play important roles in various biologic processes, such as cell differentiation, cell growth, and apoptosis^7,8^. They regulate gene expression at the post-transcriptional level by binding to the 3′-untranslated region (3′-UTR) of target messenger RNA^9^. They also participate in the development and progression of several cancers, including NSCLC^10^. Recently, studies have indicated that miRNAs could be promising targets to help diagnose or predict the prognosis of many cancers^11^. For example, serum derived miR-1246 could serve as a promising prostate cancer biomarker of cancer aggressiveness^12^. High expression of miR-146b and miR-29c in serum was significantly associated with poor 5-year overall survival (OS) in NSCLC^13^.

The PI3K/AKT/mTOR pathway is a crucial and intensively explored intracellular signaling pathway in tumorigenesis, playing a key role in regulating cellular metabolism, tumor development, growth, proliferation, metastasis, and cytoskeletal reorganization^14^. Rapamycin and its analogs (rapalogs) inhibit the function of mTOR by suppressing ribosomal S6K1 and 4E-BP1. However, the impact of rapalogs in clinical practice has been limited, as resistance to rapalogs is frequently observed upon long-term treatment^15^.

To explore the role of miRNA in NSCLC, especially in the drug response process, we used miRNA microarray analysis to detect the changes in microRNA profiles after mTOR inhibitor everolimus (RAD001) treatment. MiR-4634 was significantly upregulated in the A549 cells after RAD001 treatment. Decreased expression of miR-4634 level was also observed in NSCLC cell lines and tumors by qPCR. We also found that miR-4634 could enhance the antitumor efficacy of RAD001 by inhibiting proliferation and migration and inducing apoptosis in NSCLC cells. Furthermore, our results showed that low expression of miR-4634 was significantly associated with poor overall survival of NSCLC patients and low levels of miR-4634 could act as an independent prognostic biomarker in NSCLC. Therefore, miR-4634 could be a potential prognosis marker and therapy target, especially when combined with RAD001 in NSCLC.

## Results

### miR-4634 could be stimulated by RAD001 and related to tumorigenesis

**Figure 1:**
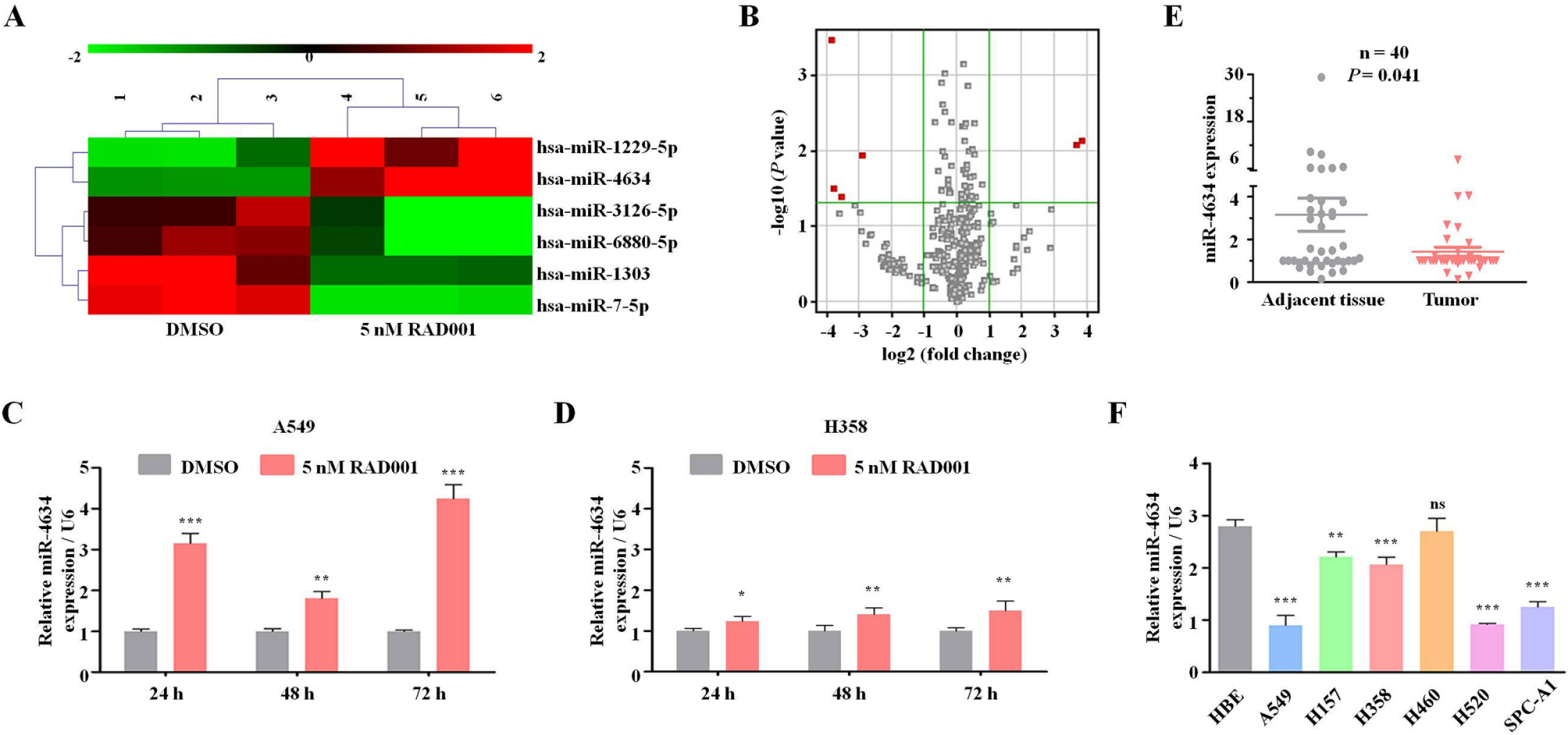
Expression of miR-4634 in NSCLC cells and tissue. (**A**) miRNA microarray analysis of six miRNAs in A549 cells treated with DMSO (control) and 5 nM RAD001. The heatmap was performed using MVE V4.9.0 (https://sourceforge.net/projects/mev-tm4/files/mev-tm4/). (**B**) Volcano plot of miRNAs from miRNA microarray. Red points indicate miRNA fold change > 2.0 and *P* < 0.05. The volcano plot was performed using Genespring software V12.0 (https://en.freedownloadmanager.org/Windows-PC/GeneSpringGX.html). (**C**, **D**) qRT-PCR was used to detect the expression of miRNA-4634 in A549 and H358 cells treated with DMSO and 5 nM RAD001 for the indicated times. The histograms in this study were performed using GraphPad Prism V5.0 (https://www.graphpad.com/). (**E**) qPCR analysis of miR-4634 performed with 40 pairs of primary NSCLC tissues and matching adjacent non-tumorous lung tissues. Statistical significance was calculated using the Wilcoxon test (*P* = 0.041). The scatter diagram was analyzed using GraphPad Prism V5.0 (https://www.graphpad.com/). (**F**) miRNA-4634 expression in HBE and NSCLC cells (A549, H157, H358, H460, H520, SPC-A1) measured with qPCR. T test. Each bar represents mean ± SD (n = 3 group). **P < 0.01, ***P < 0.001.

Using the Agilent Human miRNA chip V21.0 (Agilent Technologies Inc.), we investigated the miRNA profiles in the control group and treated group with 5 nM RAD001. After RAD001 treatment, six miRNA expressions were altered: two miRNAs (miR-1229-5p and miR-4634) increased and four miRNAs (miR-1303, miR-3126-5p, miR-6880-5p, and miR-7-5p) decreased (FC > 2.0 and *P* < 0.05) (Fig. 1A,B). Since miR-4634 dramatically increased with rarely researches now, it attracts our attentions^16^. We further confirmed miR-4634 expression after RAD001 treatment in A549 and H358 cells. qRT-PCR showed that RAD001 promotes miR-4634 expression, especially after 72 h (Fig. 1C,D). We then detected miR-4634 basal levels in NSCLC cell lines (A549, H157, H358, H520, SPC-A1) and one non-tumorigenic human bronchial epithelial (HBE) cell line. Intriguingly, we found decreased miR-4634 levels in NSCLC cell lines than in HBE cell line (Fig. 1F). 40 pairs of NSCLC specimens with matching adjacent normal tissue were collected to assess miR-4634 expression. Consistent with previous results, miR-4634 was downregulated in tumor tissue (Fig. 1E). These results showed that miR-4634 expression is reduced in NSCLC cells and can be upregulated by RAD001.

### miR-4634 has an antitumor effect and could enhance the efficacy of RAD001 in NSCLC cells

**Figure 2:**
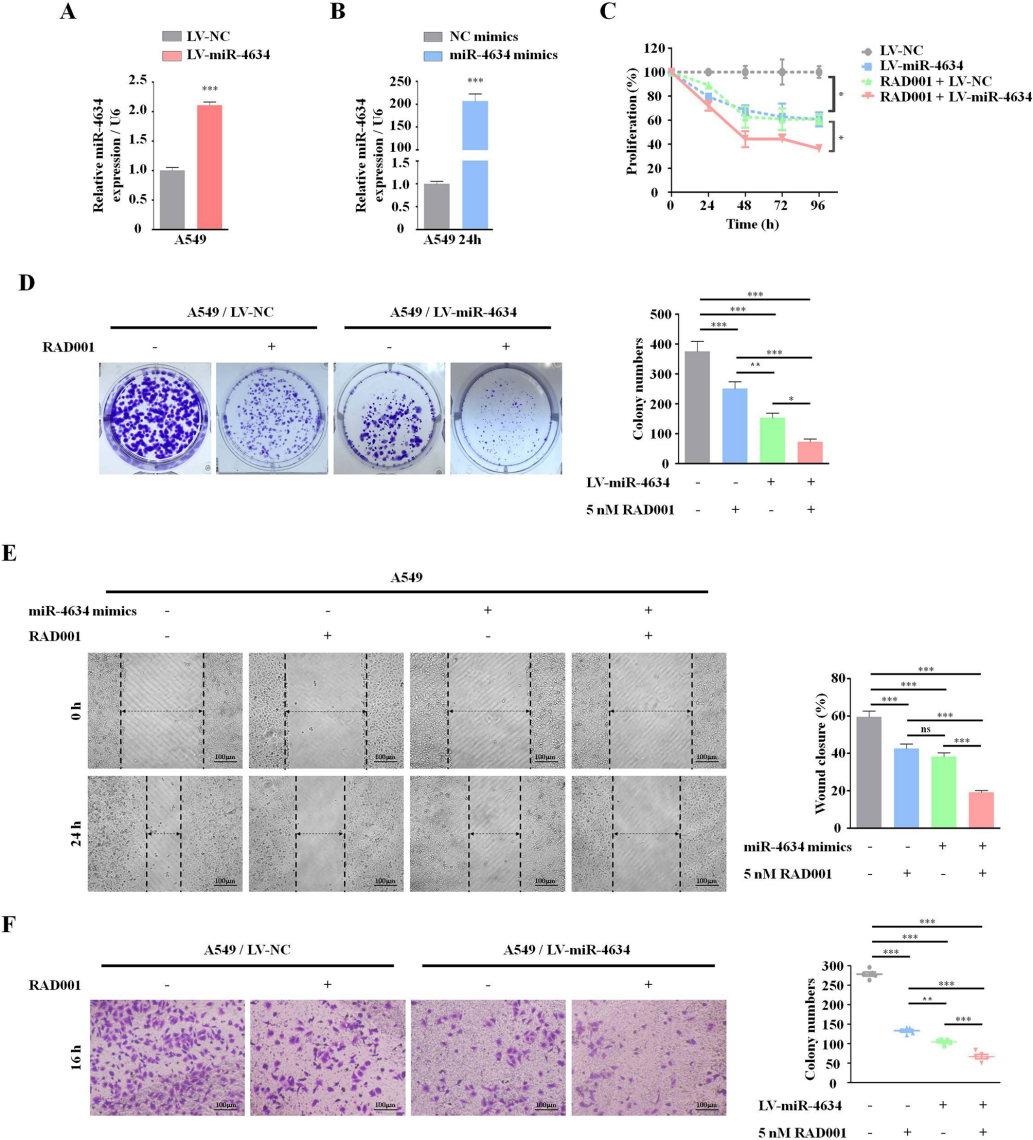
miR-4634 inhibits the proliferation and migration of A549 cells. (**A**, **B**) Cells were infected by LV-miR-4634 and transfected with miR-4634 mimics. The expression of miR-4634 was determined by qRT-PCR. All data were analyzed by the t-test. (**C**) Stable A549 cells, as indicated, were seeded in 96-well plates and then detected by CCK-8 at 0 h, 24 h, 48 h, 72 h, and 96 h. The line chart was performed using GraphPad Prism V5.0 (https://www.graphpad.com/). (**D**) Stable A549 cells were seeded in 6-well plates at a density of 1,000 cells per well. The same treatment (DMSO or 5 nM RAD001) was repeated every 3 days until day 10. The colonies in each well were counted. (**E**) Migration of A549 cells was observed at 0 h and 24 h following wounding by a pipette tip. Original magnification: 100 × . (**F**) Cellular migration ability was analyzed by transwell assay after 16 h treatment. Original magnification: × 200. Bars are means of three replicate determinations plus standard deviations. All data were analyzed by the one-way ANOVA. NS, non-significant, * *P* < 0.05, ** *P* < 0.01, ***P < 0.001.

Based on the level of miR-4634 expression, we chose A549 cell lines to investigate miR-4634 biological functions. LV-miR-4634 and miR-4634 mimics were first verified, shown in Fig. 2A,B. With increased exogenous miR-4634, cell proliferation significantly decreased, as assessed by the CCK-8 assay, and this function was enhanced by RAD001 (Fig. 2C). Furthermore, exogenous miR-4634 inhibited the colony formation ability of NSCLC cells and worked synergistically with RAD001 (Fig. 2D). Additionally, cell migration was measured by the wound-healing scratch assay, showing that miR-4634 significantly suppressed the migration ability in A549 cells. Compared to the single treatment or control group, co-treatment of miR-4634 and RAD001 showed the lowest migration rate (Fig. 2E). Similar results were observed in the transwell migration assay (Fig. 2F).

**Figure 3:**
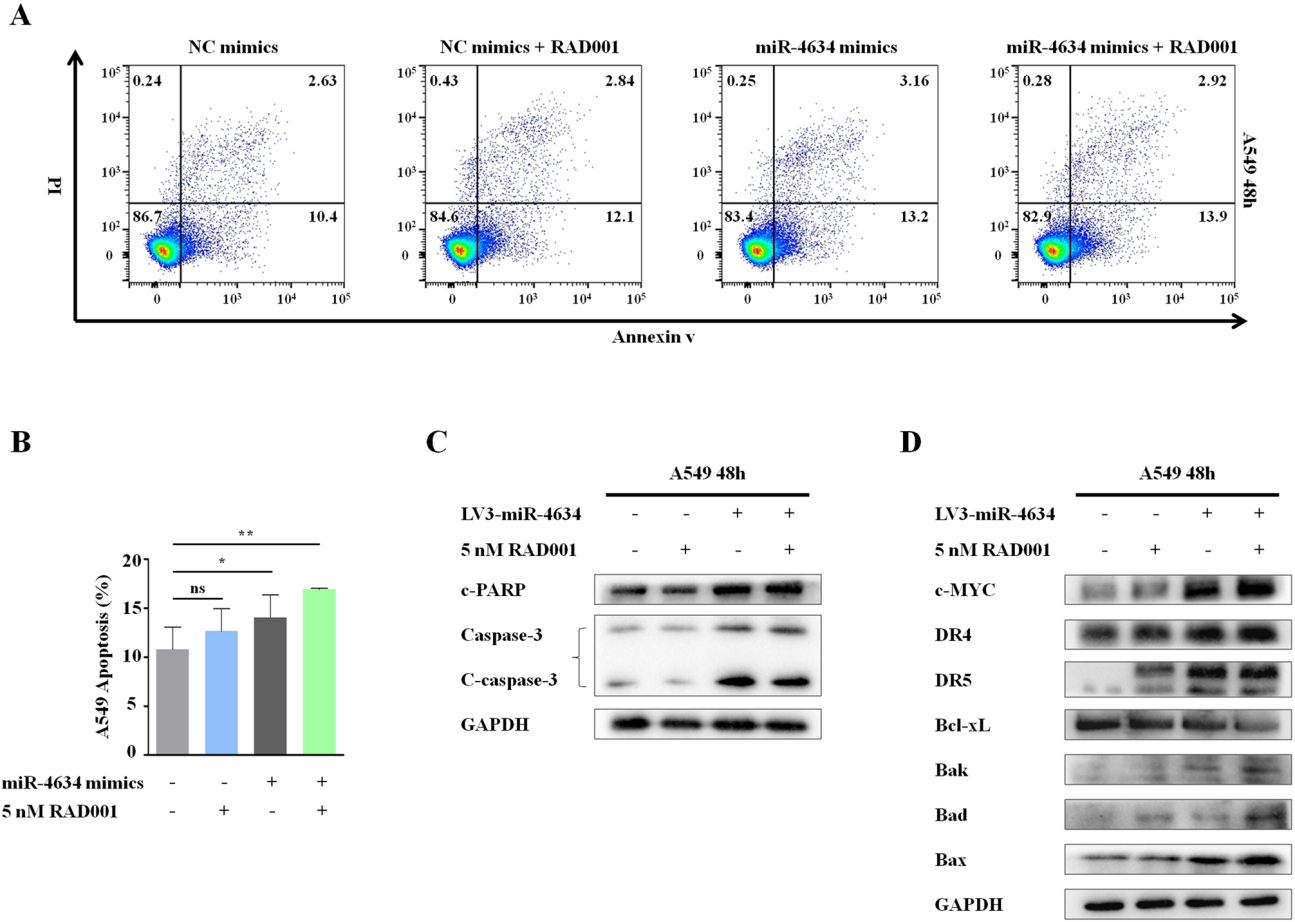
miR-4634 and RAD001 induce cell apoptosis in vitro. (**A**) A549 cells were transfected with miR-4634 mimics or NC mimics and treated with RAD001 for 48 h. Apoptotic cells were analyzed by flow cytometry using Annexin V/ PI staining. The flow diagrams were performed using FlowJo V10.0.6 (https://www.flowjo.com/solutions/flowjo/downloads/). (**B**) Each column indicates the rate of early and late apoptosis of cells, *P < 0.05, **P < 0.01. (**C**, **D**) Western blotting was used to detect the apoptosis related proteins and GAPDH was used as a loading control. The western blot analysis were performed using Image Lab V3.0 (https://en.freedownloadmanager.org/Windows-PC/Image-Lab.html). In order to save antibodies and incubate multiple antibodies at once to improve efficiency, we cut the PVDF membrane. The images of western blotting are cropped. The full-length blots/gels are presented in Supplementary Fig.S1-S11.

Flow cytometry was used to examine the effects of miR-4634 on apoptosis. Increased apoptosis was detected in A549 cells with miR-4634 mimics treatment both in the presence or absence of RAD001 when compared to the control (Fig. 3A,B). Additionally, we evaluated apoptotic molecular components c-PARP and c-caspase-3 in the A549 cells. Results showed that miR-4634 exhibited a much greater potency in inducing apoptosis (Figs. 3C, Supplementary Figs. S1–S3). To understand the mechanism of higher apoptosis induced by miR-4634 alone or combined with RAD001, key proteins known to be involved in the regulation of the extrinsic or intrinsic apoptotic pathway were detected by Western blot (Fig. 3D, Supplementary Figs. S4–S11). Results indicated that in A549 cells, the protein levels of Bad, Bak, and Bax were increased after miR-4634 transfection, while the combination of miR-4634 and RAD001 only slightly increased Bad levels. Interestingly, the expression of death receptors (DR) 5 showed similar variation. miR-4634 could enhance DR5 level without showing obvious synergistic effect with RAD001. However, there was no change in DR4 after miR-4634 or RAD001 treatment as well as with its combination compared to the control group. On the contrary, miR-4634 inhibited the expression of Bcl-xL, and the addition of RAD001 could further reduce Bcl-xL expression. These observations indicate that miR-4634 could suppress cell proliferation and migration, especially when combined with RAD001. Besides, miR-4634 promotes apoptosis via activation of both the intrinsic mitochondrial pathway and extrinsic apoptosis pathway in A549 cell lines, while the co-treatment of miR-4634 and RAD001 may induce apoptosis through the intrinsic mitochondrial pathway. In conclusion, miR-4634 could be a possible treatment target, especially when combined with RAD001.

### Associations between expression of miR-4634 and clinicopathological features of NSCLC

**Figure 4:**
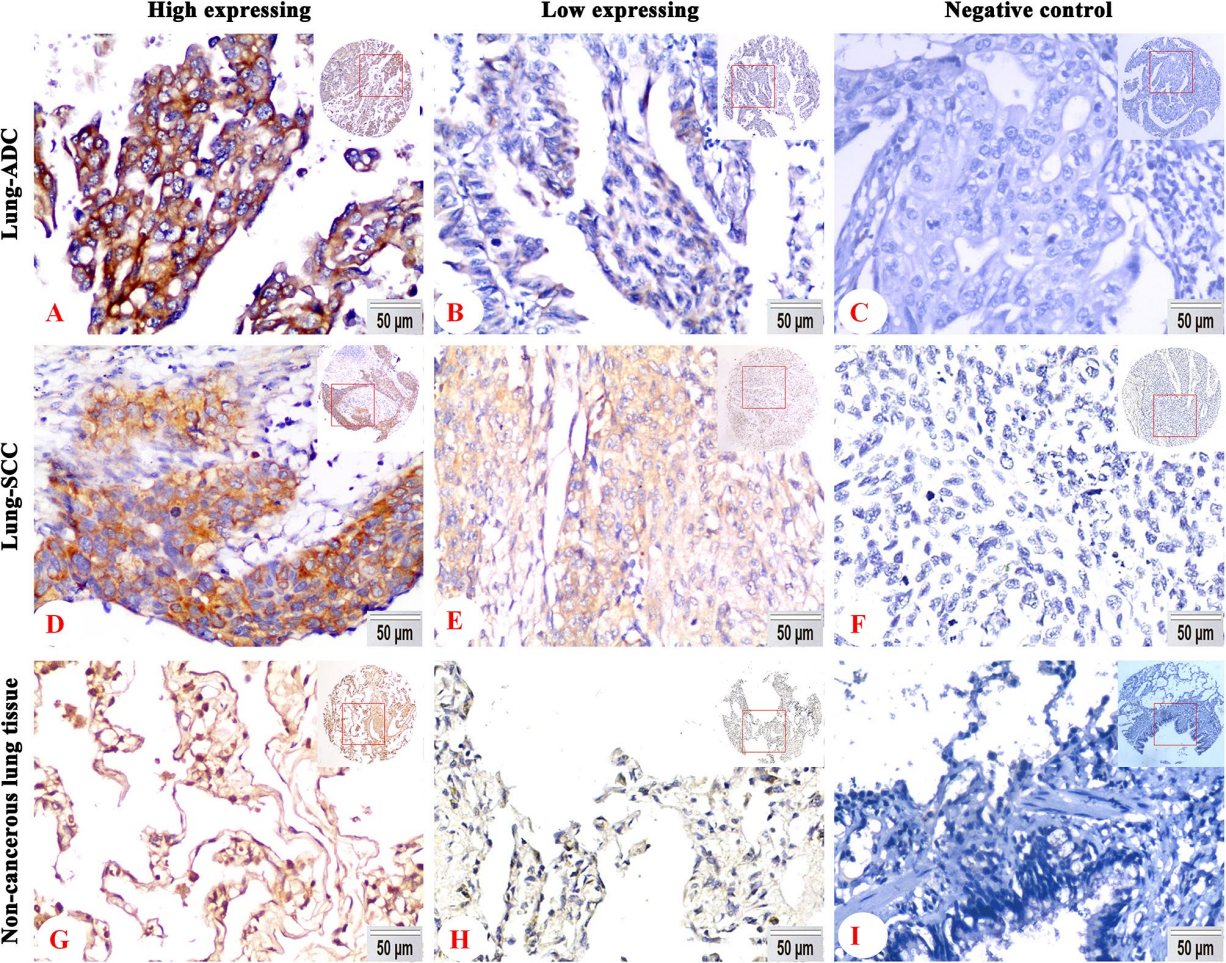
miR-4634 expression in tissue microarray of NSCLC. (**A**, **B**, **D, E**, **G**, **H**) Representative ISH staining of miR-4634 in lung SCC, ADC, and non-cancerous lung tissue using a special probe. Positive expression of miR-4634 was predominantly localized in the cytoplasm. (**C**, **F**, **I)** There was no positive signal by the control probe. (DAB staining, magnification 200 × and 40 ×).

**Figure 5:**
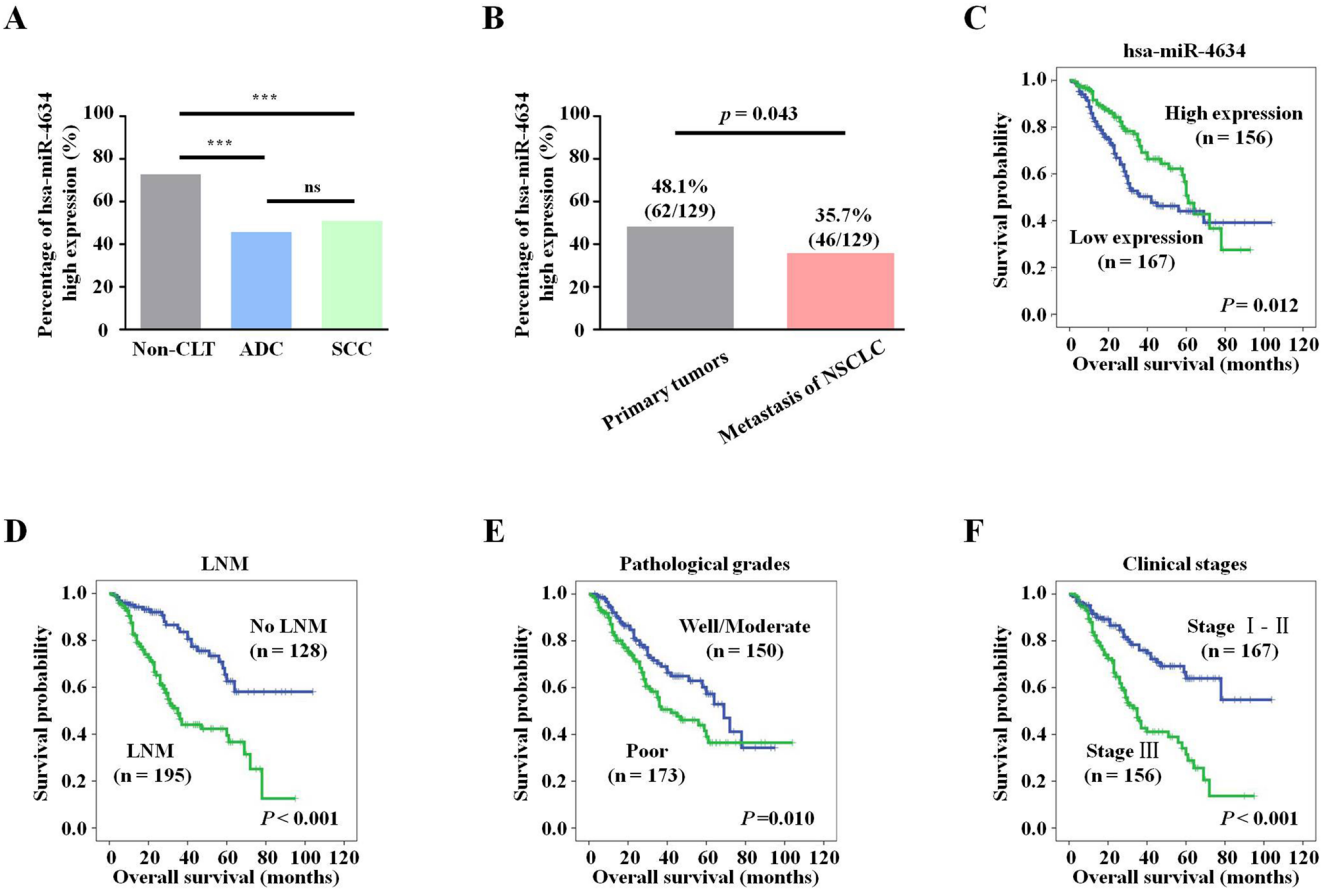
Kaplan–Meier curves for overall survival of NSCLC assessed using the log-rank test. (**A**) High expression of miR-4634 in lung SCC, ADC, and non-CLT. Significant differences were observed between the groups, statistically evaluated by chi-square test (***P < 0.001). (**B**) The high expression of miR-4634 was more frequent in primary tumors than metastasis tissue of NSCLC (*P* = 0.043). (**C**) Low expression of miR-4634 was significantly associated with short survival time of patients with NSCLC (*P* = 0.012). (**D**) Patients with LNM were more likely to have poor overall survival compared with patients without LNM (*P* < 0.001). (**E**) Patients with poorly differentiated cancer were more likely to have poorer overall survival than patients with well/moderately differentiated tumors (*P* = 0.010). (**F**) Patients with clinical stage I and II disease were more likely to have better overall survival than patients with clinical stage III tumors (*P* < 0.001). The survival curve graphs were performed using SPSS V19.0 (https://spss.en.softonic.com/).

The expression and subcellular location of miR-4634 in the NSCLC tissue microarrays (Fig. 4A–F) and noncancerous lung TMA (Fig. 4H–I) was tested by In Situ Hybridization(ISH), which contained 162 cases of lung ADC, 161 cases of lung SCC, and 92 cases of non-cancerous lung tissue. miR-4634 was specifically expressed in the cytoplasm (Fig. 4A,B,D,E) of lung ADC and lung SCC, indicating that miR-4634 may work post-transcriptionally^17^. High expression of miR-4634 was significantly more common in non-cancerous lung tissue than in ADC or SCC patients (72.8%, 45.7% and 50.9%, respectively; *P* < 0.001) (Fig. 5A).

We then collected 323 samples from NSCLC patients to analyze the associations between the expression of miR-4634 and their clinicopathological characteristics, including age, gender, histological type, lymph node metastasis (LNM) status, pathological grades, clinical stages, and survival status. Results were summarized in Table 1. Importantly, we found that high expression of miR-4634 is associated with the survival status of NSCLC patients (*P* = 0.045) (Table 1). Besides, high miR-4634 expression cases were more frequent in patients without LNM (*P* = 0.037) (Table 1). Consistent with this result, we found that the number of high miR-4634 expression was higher in primary tumors than metastasis tissues (Fig. 5B). No significant differences of miR-4634 expression were observed in age, gender, histological type, pathological grades, or clinical stages of NSCLC patients (*P* > 0.05).

**Table 1:** Analysis of the association between expression of hsa-miR-4634 and clinicopathological features of NSCLC (n = 323).

Clinicopathological features (n)	hsa-miR-4634		
	Low expression (%)	High expression (%)	*P*-value
**Age (years)**			
≤ 55 (n = 146)	68 (46.6%)	78 (53.4%)	0.094
> 55 (n = 177)	99(55.9%)	78 (44.1%)	
**Gender**			
Male (n = 240)	117 (48.8%)	123 (51.3%)	0.071
Female (n = 83)	50 (60.2%)	33 (39.8%)	
**Histological types**			
ADC (n = 162)	88 (54.3%)	74 (45.7%)	0.345
SCC (n = 161)	79 (49.1%)	82 (50.9%)	
**LNM status**			
NO LNM (n = 128)	57 (44.5%)	71 (55.5%)	0.037*
LNM (n = 195)	110 (56.4%)	85 (43.6%)	
**Differentiation**			
Well/Moderate (n = 150)	73 (48.7%)	77 (51.3%)	0.309
Poor (n = 173)	94 (54.3%)	79 (45.7%)	
**Clinical stages**			
Stage I and II (n = 167)	84 (50.3%)	83 (49.7%)	0.602
Stage III (n = 156)	83 (53.2%)	73 (46.8%)	
**Survival status**			
Alive (n = 210)	100 (47.6%)	110 (52.4%)	0.045*
Dead (n = 113)	67 (59.3%)	46 (40.7%)	

*Chi-square test, statistically significant difference (*P < 0.05).

*ADC* adenocarcinoma, *H* high expression, *L* low expression, *LNM* lymph node metastasis, *NSCLC* non-small cell lung cancer, *SCC* squamous cell carcinoma.

### High expression of miR-4634 may predict better prognosis

According to our Kaplan–Meier survival curve analysis, we found that high expression of miR-4634 is significantly associated with higher survival probability (Fig. 5C). It is worth mentioning that after 60 months, the overall survival rate of the high miR-4634 expression group showed a sharp decrease and was lower than the low expression group (Fig. 5C). This could be due to the limited sample size.

As a positive control, we also plotted the survival curve for patients with NSCLC according to the conventional prognostic parameters, including clinical stage, LNM status, and pathologic grade. As shown in Fig. 5D–F, patients without LNM, with well and moderately differentiated tumors, or with early-stage NSCLC (clinical stages I and II) had a better OS rate than patients with LNM, poor tumor differentiation, or advanced-stage NSCLC (clinical stages III), as expected (*P* < 0.001, Fig. 5D; *P* = 0.010, Fig. 5E; and *P* < 0.001, Fig. 5F, respectively). Moreover, multivariate Cox’s proportional hazard regression analysis indicated that high expression of miR-4634 could act as an independent good prognostic biomarker for NSCLC patients (*P* = 0.003), regardless of LNM status and clinical stage (*P* = 0.001, *P* = 0.001, respectively) (Table 2). However, there was no prognostic significance of patient age, gender, histological types, and pathological grades in NSCLC (*P* > 0.05 for all). In conclusion, miR-4634 could be a potential biomarker to predict NSCLC prognosis.

**Table 2:** Summary of multivariate of Cox proportional regression for overall survival in 323 cases of NSCLC.

Parameter	B	SE	Wald	Sig	Exp (B)	95.0% CI for Exp (B)	
						Lower	upper
Age	− 0.039	0.195	0.040	0.842	0.962	0.656	1.411
Gender	− 0.438	0.231	3.614	0.057	0.645	0.411	1.014
Histological types	0.247	0.201	1.518	0.218	1.281	0.864	1.898
LNM status	− 0.830	0.240	11.978	0.001***	0.436	0.272	0.698
Clinical stages	− 0.709	0.211	11.269	0.001***	0.492	0.325	0.744
Pathological grades	− 0.327	0.194	2.833	0.092	0.721	0.492	1.055
hsa-miR4634	0.397	0.197	4.061	0.044*	1.487	1.011	2.188

*CI* confidence interval; *LNM* lymph node metastasis; *NSCLC* non-small cell lung cancer.

Multivariate analysis of Cox regression, *P < 0.05, **P < 0.01, ***P < 0.001.

## Discussion

miRNAs have been attracting attention due to their unique mechanisms of function and comprehensive roles in physiological and pathological processes. Also, the utility of miRNAs in diagnosis and therapies has been introduced to various diseases, including cancers, immune diseases, and other tissue diseases^18–20^.

From our high-throughput, high-quality RNA chips, we focused on miR-4634, a rarely investigated microRNA. miR-4634 was identified as one of nine differentially expressed plasma miRNAs in rheumatoid arthritis (RA) patients. It has been shown to be significantly correlated with plasma cytokine, chemokine levels, and clinical characteristics in patients with RA and may serve as a candidate biomarker for RA diagnosis^21^. Data from Shimomura A et al. validated that a combination of serum-derived miR-4634 and other four miRNAs could distinguish breast cancer from the pancreas, biliary tract, prostate benign diseases or other cancers, with a sensitivity of 97.3%, specificity of 82.9%, and accuracy of 89.7% for breast cancer in the early stages^22^. Both human metapneumovirus (HMPV) and respiratory syncytial virus (RSV) infection can upregulate the expression of miR-4634 in human dendritic cells, which is of great significance for further understanding host–pathogen interaction between immune cells and RSV and HMPV^23^. Hitherto, the associations between the expression of miR-4634 and the clinicopathological features and prognostic implications of NSCLC have not been reported.

In our present study, we found that miR-4634 was downregulated in most NSCLC cell lines (A549, H157, H358, H520, and SPC-A1), but not in H460 cells. H460 cells express easily detectable p53 mRNA at levels comparable to normal lung tissue^24^, and we predicted that p53 may be a transcription factor of miR-4634 via the PROMO database^25^, which may be the reason for the high expression of miR-4634 in H460 cells. Significantly lower expression of miR-4634 was also observed in NSCLC clinical tumor specimens compared with the adjacent normal tissue. The ISH assay showed that the expression of miR-4634 was significantly decreased in lung ADC and SCC compared with non-cancerous lung tissues. A similar study indicating that miR-4634 is downregulated in the serum of breast cancer patients has been previously reported^22^. Importantly, high expression of miR-4634 was significantly associated with better overall survival of NSCLC patients and this might act as an independent good prognostic factor for these patients. Therefore, miR-4634 may represent a potential prognostic biomarker for NSCLC patients.

mTOR inhibitors are a Food and Drug Administration approved targeted therapy for cancer patients, including NSCLC, and is currently under extensive clinical studies^26^. RAD001 can significantly inhibit the mTORC1 pathway and enhance radiosensitivity of NSCLC cells with wild-type phosphatidylinositol-4,5-bisphosphate 3-kinase catalytic subunit alpha (PIK3CA) and Kirsten Rat Sarcoma Viral Oncogene Homolog (KRAS) by suppressing epithelial-mesenchymal transition^27^. However, long-term exposure to mTOR inhibitors induces negative feedback loops that result in protein Kinase B (Akt) activation, eukaryotic translation initiation factor 4E (eIF4E) phosphorylation, and enhancement of mTOR-targeted therapy resistance^28,29^. Therefore, an increasing number of researches have been conducted on RAD001 in combination with drugs or other molecules to improve its efficacy. For example, co-treatment with RAD001 and platycodin D, a saponin isolated from traditional Chinese herb *Platycodonis Radix*, reduced epidermal growth factor receptor and insulin growth factor 1 receptor expressions, suppressed AKT activity, reinforced 4E-BP1 inhibition, and thus terminated the feedback survival signal upon mTOR inhibition^30^. We exposed A549 cells to miR-4634 and RAD001 individually or together and observed that miR-4634 exhibited inhibitory effects on cell proliferation, colony formation, and migration in A549 cells. The combination of miR-4634 and RAD001 worked better than treatment with the individual agents. We also elucidated the mechanisms of apoptosis induction by miR-4634. Apoptosis can be triggered in a cell through either the caspase-mediated extrinsic or intrinsic pathways^31^. The extrinsic apoptotic pathway is initiated by the interaction of cell surface-exposed DRs with their respective tumor necrosis factor family ligands, such as TRAILR1 (DR4)-TRAIL and TRAILR2 (DR5)-TRAIL^32^. The intrinsic pathway is closely regulated by the B-cell lymphoma 2 (Bcl-2) family, which includes Bcl-xL (anti-apoptotic multi-domain member), Bak (pro-apoptotic multi-domain member), and Bad (pro-apoptotic BH3-only member)^33^. Our work indicates that miR-4634 decreases the expression level of Bcl-xL and upregulates Bak, Bad and Bax expression in A549 cells. DR5 expression level was also significantly increased after miR-4634 treatment. However, the expression of DR4 did not change after treatment, which may be related to long-term treatment that impairs immune surveillance of cancer cells or stimulates to escape immune surveillance^34^. More significant change of Bcl-xL level and the slight increase of Bad protein expression were observed in A549 cells treated with a combination of miR-4634 and RAD001. Furthermore, flow cytometry analysis showed that miR-4634 can improve the apoptosis effect of RAD001 in A549 cells. Based on the above results, we speculate that miR-4634 may elicit NSCLC cell apoptosis through the extrinsic apoptotic pathway and intrinsic mitochondria-mediated pathway, while its combination with RAD001 may promote apoptosis only through the intrinsic pathway. To confirm these findings, further investigations will be needed to verify the specific mechanism of miR-4634 in NSCLC; this will be the focus of our future studies. RAD001 is known to stimulate eIF4E phosphorylation and Akt activation, which is closely related to decreased cell sensitivity to RAD001 treatment and weakened anticancer efficacy of RAD001. We found that eIF4E might be the target gene of miR-4634 via TargetScanHuman 7.2^35^ (Supplementary Table S1). Thus, we hypothesize that miR-4634 probably enhances the efficacy of RAD001 by targeting 3′ UTR of eIF4E. This will be investigated further in our study.

In summary, co-treatment of miR-4634 and RAD001 exerts a synergistic inhibitory effect on cell proliferation and migration, as well as inducing apoptosis via activation of extrinsic apoptotic pathway and intrinsic mitochondrial-mediated pathway. miR-4634 might be a novel therapeutic strategy to augment the efficacy of RAD001 therapy in NSCLC. Furthermore, high expression of miR-4634 might be an independent good prognostic biomarker for NSCLC.

## Materials and methods

### Cell line and cell culture

The human NSCLC line A549 was obtained from the Cell Bank of the Chinese Academy of Sciences (Shanghai, China) where they were characterized by mycoplasma detection, DNA-Fingerprinting, isozyme detection and cell vitality detection. A549 cell line was immediately cultured and frozen down such that the cell line could be restarted every 3–4 months from a frozen vial of the same batch of cells. All cells were cultured in RPMI-1640 (Gibco, USA) medium with 10% fetal bovine (FBS) (Gibco, USA) at 37 °C in a humidified 5% CO_2_ incubator^28^.

### Reagents and antibodies

The synthetic miRNA probe miR-4634 (#YD00611033) (QIAGEN, Germany), was resuspended in RNase-free water at 25 µM, probe sequence 5′-CGGGCCGGTCGCGC-3′. RAD001 (Selleckchem, Houston, TX, USA). The primary antibody anti-GAPDH mAb (60,004–1-Ig) at a 1:50,000 dilutions was purchased from Proteintech Group. Other primary antibodies were purchased from Cell Signaling Technology, such as cleaved-PARP (#5625) at a 1:1,000 dilutions, DR4 (#42,533) at a 1:1,000 dilutions, DR5 (#8,074) at a 1:1,000 dilutions, c-Myc (#18583) at a 1:1,000 dilutions, Bcl-xL (#2764) at a 1:1,000 dilutions, Bak (#12105) at a 1:1,000 dilutions, Bax (#5023) at a 1:1,000 dilutions, Bad (#9239) at a 1:1,000 dilutions.

### miRNA microarray analysis

Cells were plated at 1 × 10^5^ cells/well in triplicate cell culture plates and treated with vehicle or 5 nM RAD001 the next day. Total RNA were extracted using TRIzol reagent (Invitrogen) after 24 h treatment. Concentration and purity of the total RNA were assessed with NanoDrop ND-2000 (Thermo Scientific) and RNA integrity was verified using Agilent 2100 Bioanalyzer (Agilent Technologies). Total RNA were dephosphorylated, denatured and then labeled with Cyanine-3-CTP. The Oebiotech company performed the miRNA microarray assay. The labeled RNA sample was competitively hybridized to a miRNA microarray (Agilent Human miRNA microarray Design ID: 070156). After washing, the arrays were scanned with the Agilent Scanner G2505C (Agilent Technologies).

Feature Extraction software (version10.7.1.1, Agilent Technologies) was used to analyze array images to get raw data. Next, Genespring software (version 12.5; Agilent Technologies) was employed to finish the basic analysis with the raw data. To begin with, the raw data was normalized with the quantile algorithm. At least one set of probes with 100% labeled as “Detected” was chosen for subsequent analysis. Differentially expressed miRNAs were then identified through fold change as well as P value calculated using t-test. The threshold set for up- and down-regulated genes was a fold change (FC) ≥ 2.0 and a P value ≤ 0.05. Target genes of differentially expressed miRNAs were the intersection predicted with Targetscan databases^35^.

### Quantitative RT-PCR analysis

Total RNA were extracted using TRIzol reagent (Invitrogen) and 2 μg of total RNA was used for synthesis of Mir-X miRNA First-Strand Synthesis Kit (Takara). The miRNA level was measured by qPCR with SYBR Advantage qPCR Premix (Takara) on CFX96 Real-Time PCR Detection System (Bio-Rad). Relative expression was determined with a U6 control through the 2−ΔΔ Ct method.

Forward primers were 5′-CGGCGCGACCGGCCCGGGG-3′ (miR-4634), a universal primer against the stem-loop region 5′-GTGCAGGGTCCGAGGT-3′.

### MiRNAs mimic and transfection

The synthetic miR-4634 mimics and NC mimics (GenePharma, Shanghai, China), were resuspended in DEPC water at 20 µM. Briefly, NC mimics, miR-4634 mimics and Lipofectamine 3,000 were diluted with 100 µl serum-free medium, respectively. The diluted Lipofectamine 3,000 (Invitrogen, USA) was added into the diluted NC mimics or miR-4634 mimics, respectively, and incubated for 30 min at room temperature, following which they were added to the cell suspension.

### Lentivirus infection

The miR-4634 overexpression lentivirus LV-miR-4634 and its control virus LV-NC were purchased from GenePharma (Shanghai, China). To generate stably transduced cell lines, A549 cells were infected with LV-NC or LV-miR-4634 for 2 days, and then subjected to puromycin selection (2.5 mg/mL) for 2 weeks.

### Cell proliferation assay

Suspension of stable A549 cells was plated in 96-well plates and grown at 37 °C. Then, the cells were treated with dimethyl sulfoxide (DMSO) or 5 nM RAD001 for 0 h, 24 h, 48 h, 72 h, and 96 h. After treatment, the culture medium was removed. 90 μL fresh medium and 10 μL Cell Counting Kit-8 (CCK-8) (Dojindo, Japan) were added to each well. The plates were incubated for 3 h in the 37 °C incubator. The absorbance at 450 nm was measured on an automated reader.

### Colony formation assay

The colony formation assay was performed as previously described^36^.

### Wound healing assays

A total of 1 × 10^5^ growing cells were seeded into a 6-well plate and incubated for 24 h at 37 °C. A549 cells were scratched at 24 h after transfection and washed thrice with phosphate buffered saline (PBS). Then fresh culture medium without FBS was added and treated to 5 nM RAD001 or DMSO. The size of wounds was observed and images were captured using inverted microscope (Leica, Germany) at 0 h and 24 h.

### Transwell migration assays

Transwell migration assays were performed using a 24-well chamber (Costar 3422; Corning Inc., Corning, NY, USA). A549 cells (2 × 104) were suspended in 100 μl of serum-free medium in the upper chamber. Medium containing 10% FBS was added to the lower chamber. The cells could migrate for 24 h to allow cell migration through the membrane. The membrane was fixed in 4% paraformaldehyde for 30 min and stained with crystal violet. Cells on the lower side of the membrane were counted^37^.

### Detection of cell apoptosis

Detection and analysis of apoptosis by flow cytometry were performed as previously described^28^.

### Western blotting

Cells were lysed in RIPA buffer (Beyotime, China) containing protease inhibitor and phosphatase inhibitors (AURGENE, China). The protein lysates were boiled in 5 × SDS buffer. Proteins were separated on SDS polyacrylamide gel electrophoresis (PAGE) and transferred to polyvinylidene difluoride (PVDF) membrane (Millipore, USA) for blotting with antibodies. Antibody-antigen binding was detected by the enhanced chemiluminescence (ECL) system.

### Clinical data

In this study, 337 cases of NSCLC were submitted to surgical treatment at the Department of Thoracic Surgery at the Second Xiangya Hospital of Central South University (Changsha, China) from 2003 to 2013. 92 cases of non-cancerous lung tissues were chosen randomly from the files of the Department of Pathology at the Second Xiangya Hospital of Central South University (Changsha, China). Besides, 40 pairs of primary non-small cell lung cancer tissues and adjacent non-tumor lung tissues were collected from 40 cases of NSCLC. These cases were submitted to surgical treatment at the Department of Thoracic Surgery at the Second Xiangya Hospital of Central South University (Changsha, China) in 2019. All NSCLC patients had been submitted to routine staging examination and effectively treated by surgical resection of both the primary tumor (at least lobectomy) and systematic mediastinal lymph node dissection. These patients had a confirmed histological diagnosis of NSCLC according to World Health Organization histological classification of the lung cancer. The staging classification of the current analysis was carried out based on the criteria of the 8th edition of the AJCC/UICC TNM staging system of lung cancer (2017). Patients had not been treated with chemotherapy or radiotherapy before the original operation. Complete clinical record and follow-up data were available for all patients^27^.

### ISH and scores

The staining condition for miR-4634 probe was adjusted according to our laboratory experience. We used the tissue microarrays (TMA) technology designed and constructed high-throughput NSCLC TMAs according to rules previously described^38^. A series of 4-μm TMA sections were applied to ISH analysis. Briefly, each TMA section was deparaffinized in turpentine, rehydrated on alcohol at a descending scale and digested with Proteinase-K (TIB, Xiamen, China) diluted 1: 25, for 1 min, at room temperature. The digestion for all samples was terminated in Tris–HCl buffered Saline + Tween (TBST) and pre-hybridized with the probe mixture for an hour at 55 °C, then hybridized at 37 °C overnight. Sections were incubated with three antibodies consecutively, which was added according to the manufacturer's instructions. Color reaction was developed by using 3, 3′-diaminobenzidine tetrachloride chromogen solution. All slides were counterstained with hematoxylin^39^. The specificity of the antibody was determined without probe as a negative control.

ISH staining of TMA sections was scored independently evaluated under the light microscope at a magnification of × 200 by two independent pathologists Qiuyuan Wen and Yuting Zhan who blinded to the clinical data. The scores were based on the staining intensity and extent of staining^40^. Staining intensity for miR-4634 was scored as 0 (negative), 1 (weak), 2 (moderate), and 3 (strong) and staining extent was scored as 0 (0%), 1 (1–25%), 2 (26–50%), 3 (51–75%), and 4 (76–100%). Overall score = intensity score × extent score^41^. The total score ranged from 0 to 12, and the score of 0 to 6 was defined as low expression, therefore 7 to 12 were considered high expression. Agreement between the 2 evaluators was 95%, and all scoring discrepancies were resolved through discussion^42^.

### Statistical analysis

All statistical analyses were performed using SPSS version 19 (SPSS, Chicago, IL). The relationship between the expression of miR-4634 and clinicopathological features of NSCLC was analyzed by the chi-square test. Kaplan–Meier analysis was performed for OS curves and statistical significance was assessed using the log-rank test. To estimate whether miR-4634 was an independent prognostic factor, the Cox proportional hazard regression model was used. Other statistical significances were determined by the One-way ANOVA test and the Dunnett-T3-test especially for cell colonies. Two-sided statistical analysis was used and P < 0.05 was considered to be statistically significant.

### Ethics statement

All samples were obtained with informed consent and all protocols were approved by the Second Xiangya Hospital of Central South University Ethics Review Board (Scientific and Research Ethics Committee, No. S039/2011). Also, written informed consent was taken from all patients involved in our study. All methods were carried out in accordance with relevant guidelines and regulations.

## Supplementary information

Supplementary file1 (DOCX 5779 kb)

Supplementary file2 (XLSX 66 kb)

## Data Availability

The authors declare that all available data are present in the manuscript and Supplementary Files.

## References

[1] Cao M, Chen W (2019). Epidemiology of lung cancer in China. Thorac. Cancer.

[2] Siegel RL, Miller KD, Jemal A (2019). Cancer statistics, 2019. CA Cancer J. Clin..

[3] Feng RM, Zong YN, Cao SM, Xu RH (2019). Current cancer situation in China: good or bad news from the 2018 Global Cancer Statistics?. Cancer Commun. (Lond).

[4] Bica-Pop C (2018). Overview upon miR-21 in lung cancer: focus on NSCLC. Cell Mol. Life Sci..

[5] Jin B, Jin H, Wu HB, Xu JJ, Li B (2018). Long non-coding RNA SNHG15 promotes CDK14 expression via miR-486 to accelerate non-small cell lung cancer cells progression and metastasis. J. Cell. Physiol..

[6] Xiao Y (2018). FBXW7 deletion contributes to lung tumor development and confers resistance to gefitinib therapy. Mol. Oncol..

[7] Moro M (2019). Coated cationic lipid-nanoparticles entrapping miR-660 inhibit tumor growth in patient-derived xenografts lung cancer models. J. Control Release.

[8] Zhao L (2018). MiroRNA-188 acts as tumor suppressor in non-small-cell lung cancer by targeting MAP3K3. Mol. Pharm..

[9] Zang H, Wang W, Fan S (2017). The role of microRNAs in resistance to targeted treatments of non-small cell lung cancer. Cancer Chemother. Pharmacol..

[10] Yu X (2018). miR-195 targets cyclin D3 and survivin to modulate the tumorigenesis of non-small cell lung cancer. Cell Death Dis..

[11] Khandelwal A (2019). Circulating microRNA-590-5p acts as a liquid biopsy marker in non-small cell lung cancer. Cancer Sci..

[12] Bhagirath D (2018). microRNA-1246 is an exosomal biomarker for aggressive prostate cancer. Cancer Res..

[13] Yang X (2019). Serum microRNA signature is capable of early diagnosis for non-small cell lung cancer. Int. J. Biol. Sci..

[14] Polivka J, Janku F (2014). Molecular targets for cancer therapy in the PI3K/AKT/mTOR pathway. Pharmacol. Ther..

[15] He L (2019). Regulation of GSK3 cellular location by FRAT modulates mTORC1-dependent cell growth and sensitivity to rapamycin. Proc. Natl. Acad. Sci. USA.

[16] Yang P (2019). Endometrial miR-543 is downregulated during the implantation window in women with endometriosis-related infertility. Reprod. Sci..

[17] Dragomir MP, Knutsen E, Calin GA (2018). SnapShot: unconventional miRNA functions. Cell.

[18] Zhang H (2019). Electrochemiluminescence-microscopy for microRNA imaging in single cancer cell combined with chemotherapy-photothermal therapy. Anal. Chem..

[19] Chen W (2019). Decreased expression of mitochondrial miR-5787 contributes to chemoresistance by reprogramming glucose metabolism and inhibiting MT-CO3 translation. Theranostics.

[20] Acharya A (2019). miR-26 suppresses adipocyte progenitor differentiation and fat production by targeting Fbxl19. Genes Dev..

[21] Wang W (2015). Plasma microRNA expression profiles in Chinese patients with rheumatoid arthritis. Oncotarget.

[22] Shimomura A (2016). Novel combination of serum microRNA for detecting breast cancer in the early stage. Cancer Sci..

[23] Banos-Lara MDR, Zabaleta J, Garai J, Baddoo M, Guerrero-Plata A (2018). Comparative analysis of miRNA profile in human dendritic cells infected with respiratory syncytial virus and human metapneumovirus. BMC Res. Notes.

[24] Takahashi T (1989). p53: a frequent target for genetic abnormalities in lung cancer. Science.

[25] Messeguer X (2002). PROMO: detection of known transcription regulatory elements using species-tailored searches. Bioinformatics.

[26] Orr-Asman MA (2017). mTOR kinase inhibition effectively decreases progression of a subset of neuroendocrine tumors that progress on rapalog therapy and delays cardiac impairment. Mol. Cancer Ther..

[27] Chen Y (2019). mTORC1 inhibitor RAD001 (everolimus) enhances non-small cell lung cancer cell radiosensitivity in vitro via suppressing epithelial-mesenchymal transition. Acta Pharmacol. Sin..

[28] Wen Q (2016). CGP57380 enhances efficacy of RAD001 in non-small cell lung cancer through abrogating mTOR inhibition-induced phosphorylation of eIF4E and activating mitochondrial apoptotic pathway. Oncotarget.

[29] Rodrik-Outmezguine VS (2011). mTOR kinase inhibition causes feedback-dependent biphasic regulation of AKT signaling. Cancer Discov..

[30] Li T (2017). Novel Hsp90 inhibitor platycodin D disrupts Hsp90/Cdc37 complex and enhances the anticancer effect of mTOR inhibitor. Toxicol. Appl. Pharmacol..

[31] Wong RS (2011). Apoptosis in cancer: from pathogenesis to treatment. J. Exp. Clin. Cancer Res..

[32] Guicciardi ME, Gores GJ (2009). Life and death by death receptors. FASEB J..

[33] Pistritto G, Trisciuoglio D, Ceci C, Garufi A, D'Orazi G (2016). Apoptosis as anticancer mechanism: function and dysfunction of its modulators and targeted therapeutic strategies. Aging (Albany NY).

[34] Yao W (2020). Correction: Expression of death receptor 4 is positively regulated by MEK/ERK/AP-1 signaling and suppressed upon MEK inhibition. J. Biol. Chem..

[35] Agarwal V, Bell GW, Nam JW, Bartel DP (2015). Predicting effective microRNA target sites in mammalian mRNAs. Elife.

[36] Wang W (2017). Suppression of beta-catenin nuclear translocation by CGP57380 decelerates poor progression and potentiates radiation-induced apoptosis in nasopharyngeal carcinoma. Theranostics.

[37] Zhang X (2019). Hypoxic BMSC-derived exosomal miRNAs promote metastasis of lung cancer cells via STAT3-induced EMT. Mol. Cancer.

[38] Chu S (2017). High expression of heat shock protein 10 correlates negatively with estrogen/progesterone receptor status and predicts poor prognosis in invasive ductal breast carcinoma. Hum. Pathol..

[39] Wen Q (2015). Flot-2 expression correlates with EGFR levels and poor prognosis in surgically resected non-small cell lung cancer. PLoS ONE.

[40] Mou K (2016). Relationship between miR-7 expression and treatment outcomes with gefitinib in non-small cell lung cancer. Oncol. Lett..

[41] Zheng H (2019). Elevated expression of G3BP1 associates with YB1 and p-AKT and predicts poor prognosis in nonsmall cell lung cancer patients after surgical resection. Cancer Med..

[42] Zheng H (2019). Expression of DR5 and cFLIP proteins as novel prognostic biomarkers for nonsmall cell lung cancer patients treated with surgical resection and chemotherapy. Oncol. Rep..

