# Supplementary material for: miR-4634 augments the anti-tumor effects of RAD001 and associates well with clinical prognosis of non-small cell lung cancer
Source: Sci Rep. 2020 Aug 4;10:13079. doi: 10.1038/s41598-020-70157-0 (PMC7403585; doi:10.1038/s41598-020-70157-0)
Supplement: Supplementary file 1 — Supplementary file1 (DOCX 5779 kb) [file 41598_2020_70157_MOESM1_ESM.docx]

**miR-4634** **augments the anti-tumor effects of RAD001 and associates well with clinical prognosis of non-small cell lung cancer**

Sile Liu^1^, Hongjing Zang^1^_,_ Hongmei Zheng^1^, Weiyuan Wang ^2^, Qiuyuan Wen^1^, Yuting Zhan^1^, Yang Yang^1^, Yue Ning^1^, Haihua Wang^1^, Songqing Fan PhD^1^*

1. Department of Pathology, the Second Xiangya Hospital, Central South University, Changsha, Hunan, 410011, China

2. Department of Pathology, Xiangya Hospital of Central South University, Changsha, 410011, Hunan, China


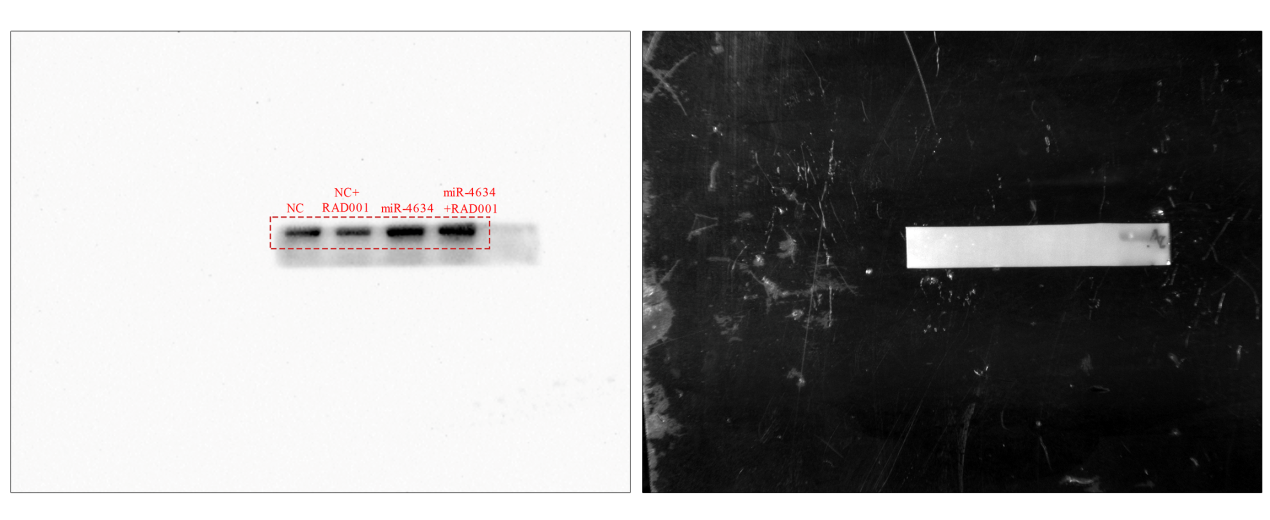


**Figure S1.** The raw western blotting results of c-parp.


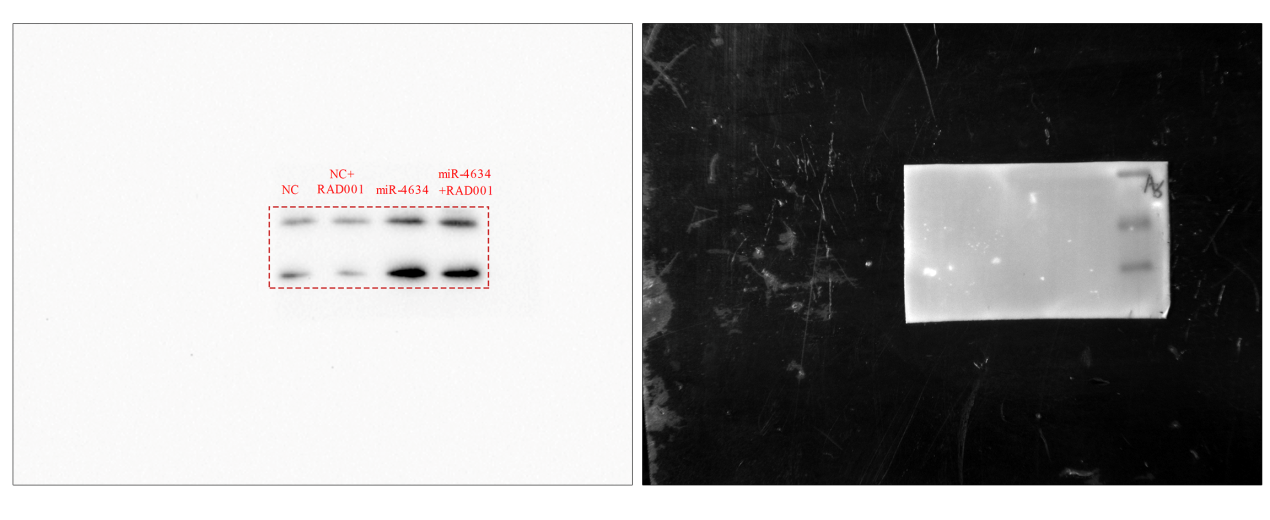


**Figure S2.** The raw western blotting results of caspase-3.


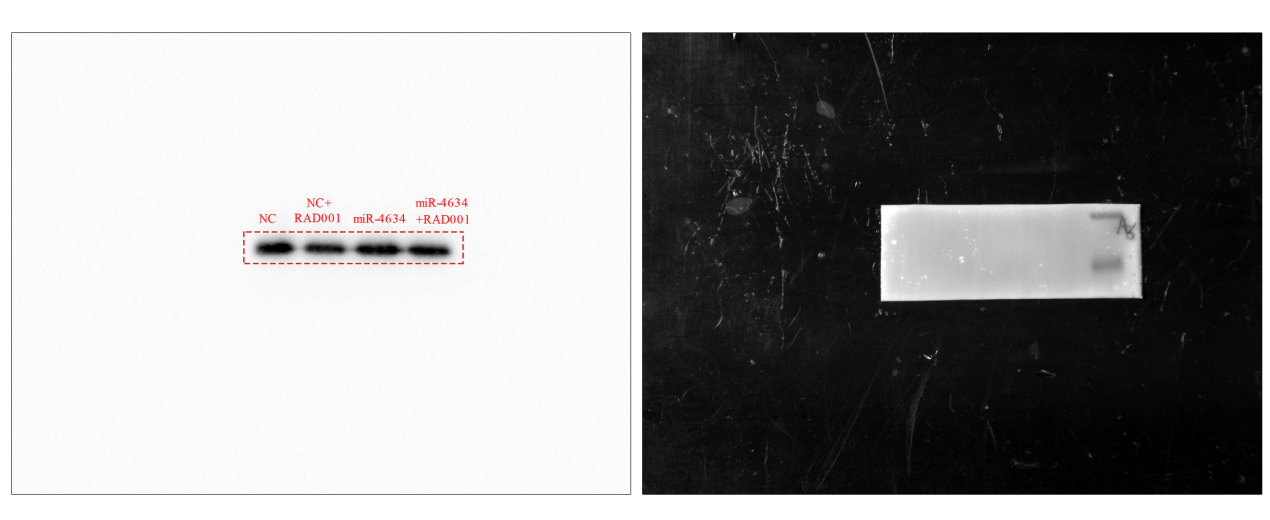


**Figure S3.** The raw western blotting results of GAPDH.


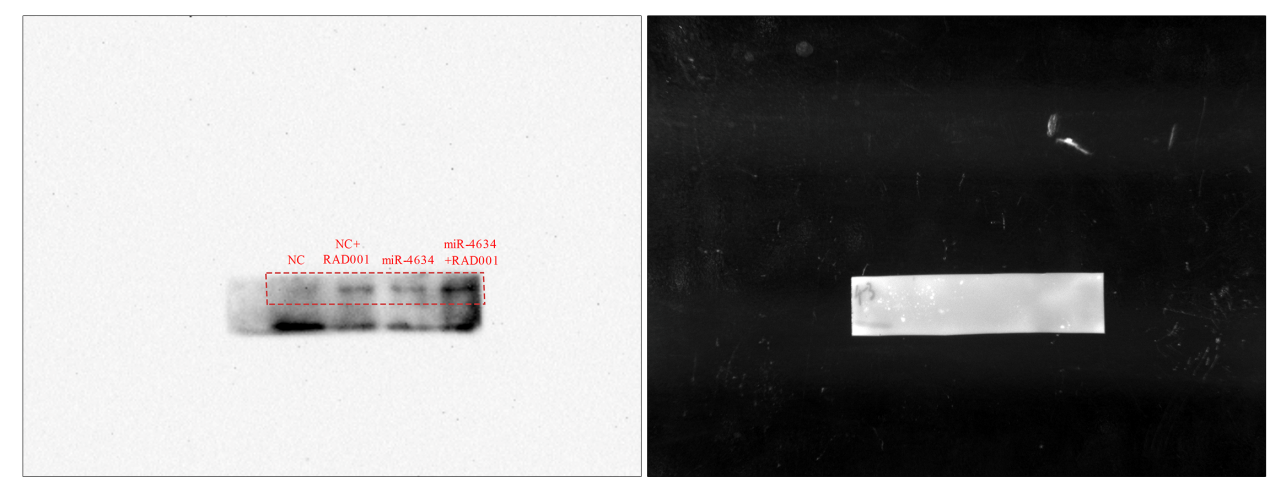


**Figure S4.** The raw western blotting results of Bad.


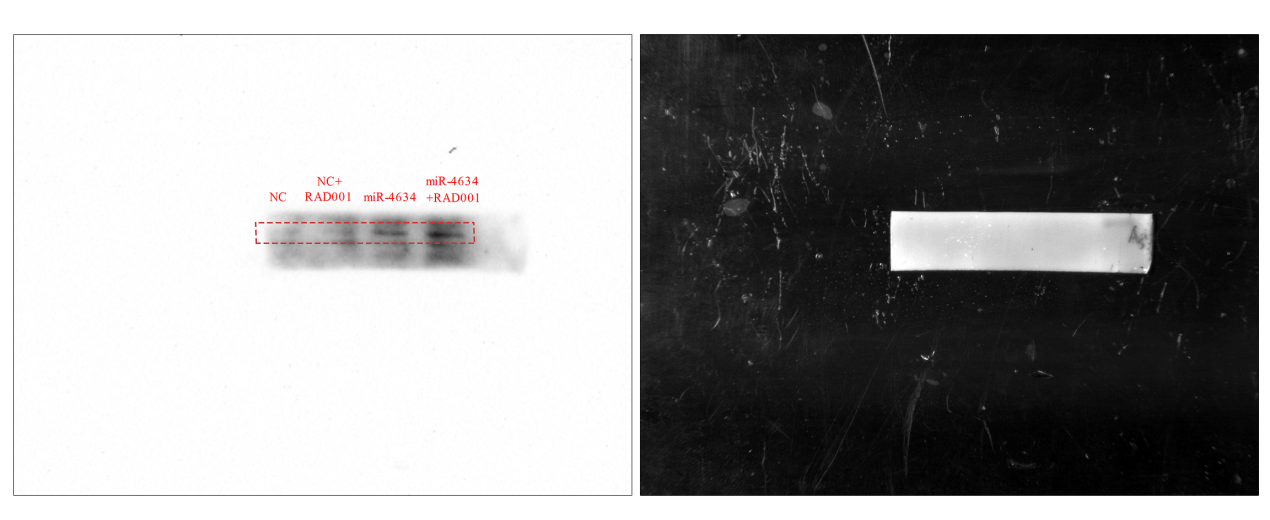


**Figure S5.** The raw western blotting results of Bak.


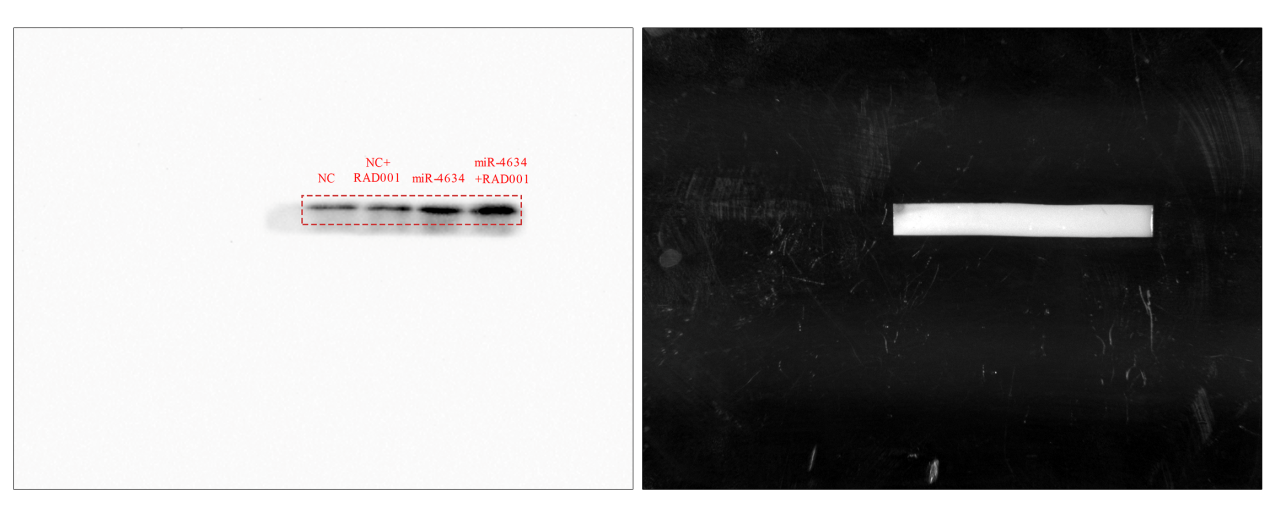


**Figure S6.** The raw western blotting results of Bax.


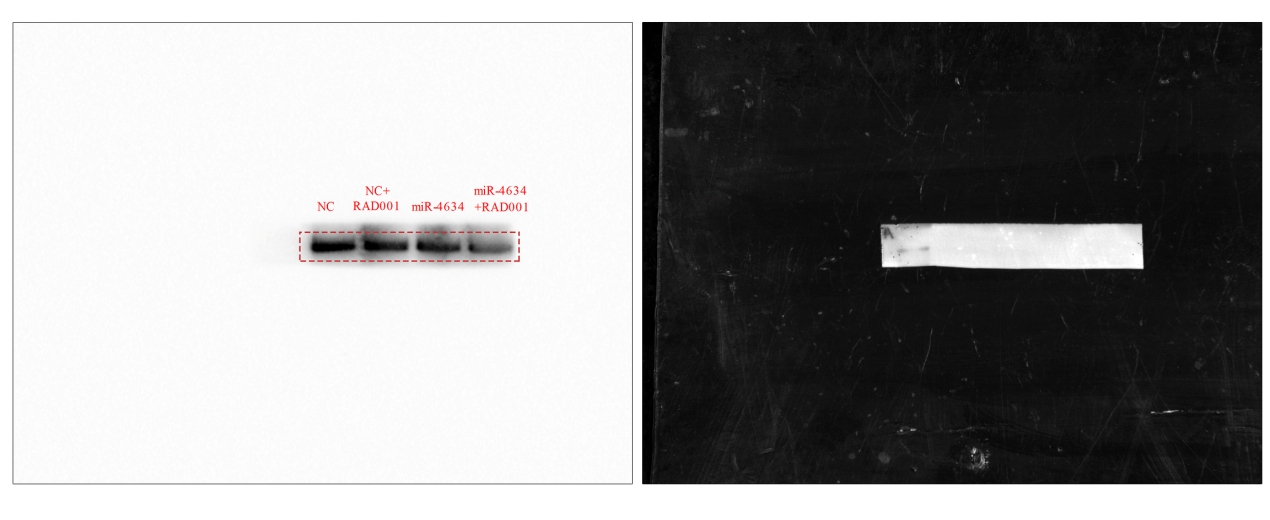


**Figure S7.** The raw western blotting results of Bcl-xL.


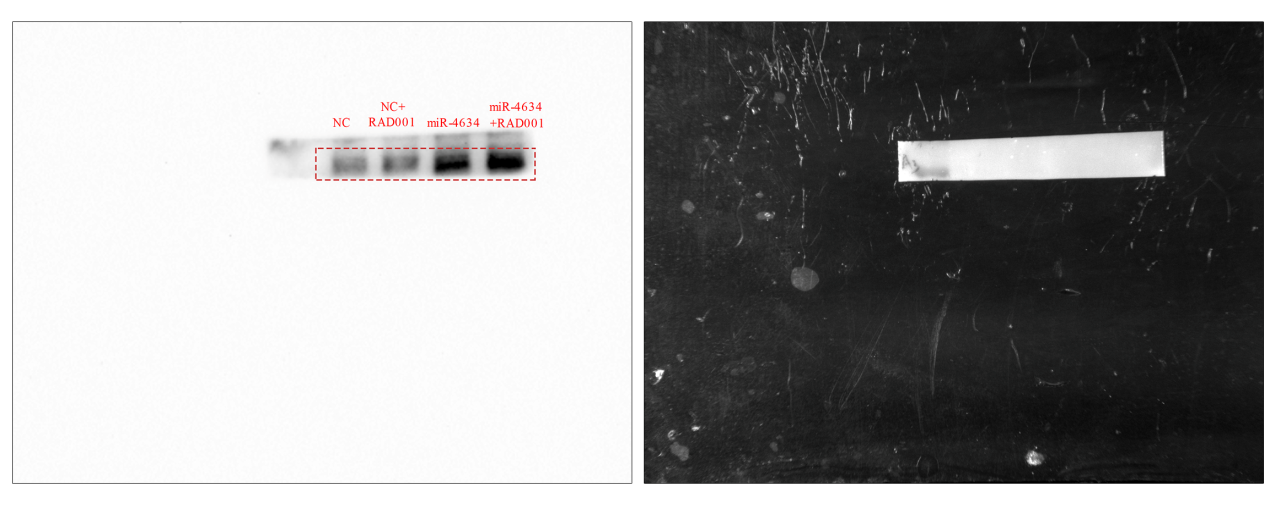


**Figure S8.** The raw western blotting results of c-myc.


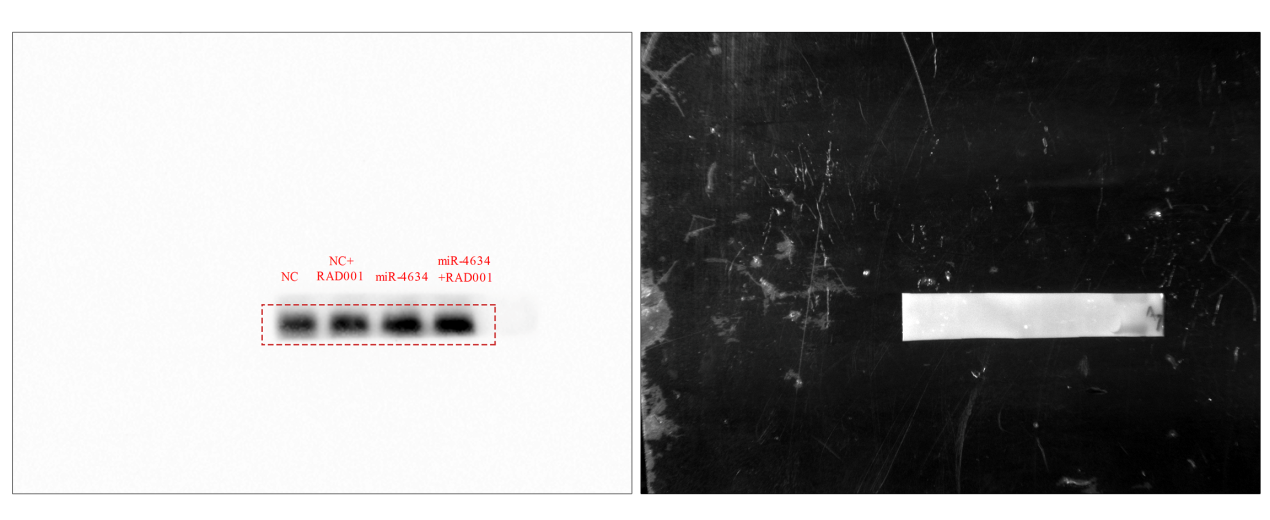


**Figure S9.** The raw western blotting results of DR4.


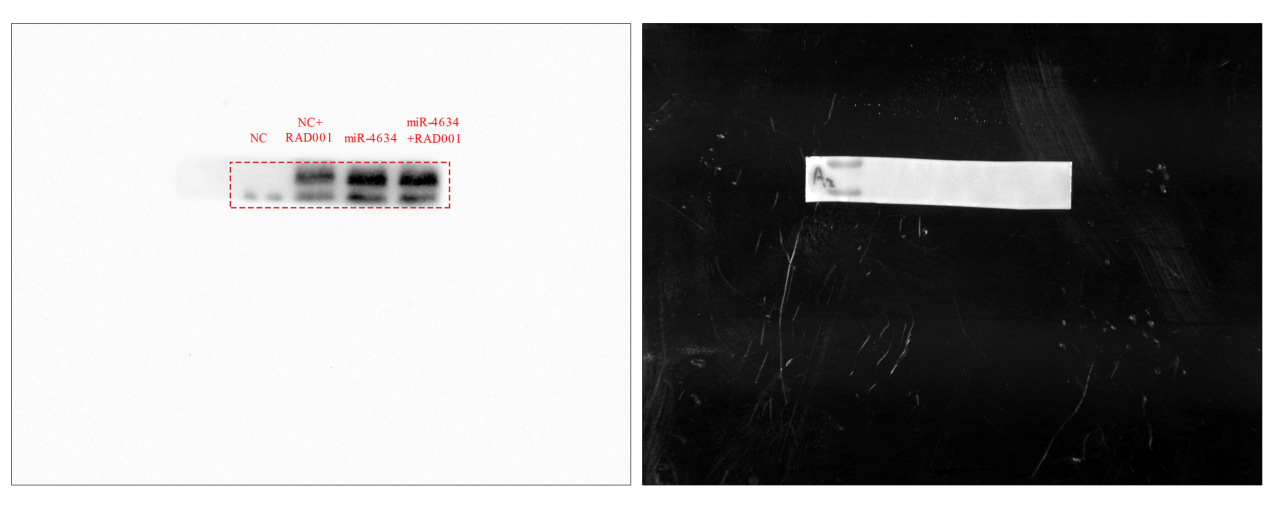


**Figure S10.** The raw western blotting results of DR5.


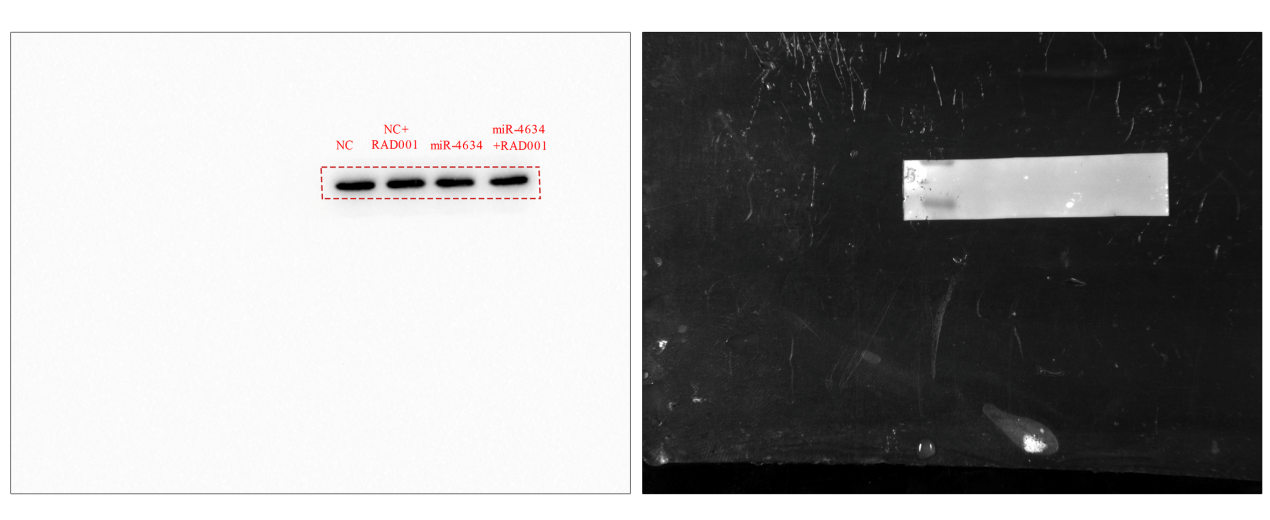


**Figure S11.** The raw western blotting results of GAPDH.

**Table S1**. Target genes of miR-7-5p was predicted with Targetscan databases.
